# Unpacking the Relationship Between Social Engagement and Cognitive Decline: A Longitudinal Analysis of Structural, Functional, and Qualitative Dimensions

**DOI:** 10.1002/alz70860_099579

**Published:** 2025-12-23

**Authors:** Nicole Ee, Fiona E Matthews, Ruth Peters, Hamidul Huque, Kaarin J. Anstey

**Affiliations:** ^1^ The George Institute for Global Health, Sydney, NSW, Australia; ^2^ University of New South Wales, Sydney, NSW, Australia; ^3^ Neuroscience Research Australia, Sydney, NSW, Australia; ^4^ University of Hull, Hull, United Kingdom; ^5^ The George Institute for Global Health, University of New South Wales, Imperial College London, Sydney, NSW, Australia; ^6^ UNSW Ageing Futures Institute, Syndey, NSW, Australia; ^7^ Neuroscience Research Australia (NeuRA), Sydney, NSW, Australia

## Abstract

**Background:**

Social engagement is a well‐recognized protective factor against cognitive decline and dementia. However, the specific roles of its structural (e.g. network size or interaction frequency), functional (e.g. emotional or instrumental support), and qualitative (e.g. relationship quality) dimensions remain unclear. Heterogeneity in the operationalization of social engagement across the literature has further complicated efforts to elucidate clear risk and protective pathways linking these aspects to cognitive health.

**Method:**

Data were drawn from 1531 participants aged 68–72 years (M=70.56, SD=1.48) across two waves (4 years apart) of the Personality and Total Health Through Life study, a representative, community‐based cohort originally from Canberra and surrounds in Australia. Factor analysis identified the constructs underlying 29 observed measures of social engagement. Latent Variable Path Models, adjusted for age, sex, and education, were employed to examine associations between the identified factors and cognitive change across five validated measures covering memory, processing speed (PS), and executive function (EF).

**Result:**

Factor analysis identified eight factors; networks of family and friends, positive relationships with family and friends (structure, function, and quality), positive partner relationships (function and quality), and negative relationships with partners, friends, and family (structure and quality). None uniquely measured structural, functional, or qualitative engagement. Negative relationships with partners and friends were associated with slower declines in EF (β=‐2.46 to ‐4.97, *p* < .05), memory (β=1.24, *p* < .05), and *p*s (β=4.52, *p* < .05), whereas negative family relationships were linked to greater declines across all cognitive domains (β_memory_=‐1.59 to ‐1.80, β_PS_=5.94, β_EF_=4.45 to 6.21, *p* < .05). Primarily structural factors (social networks) and all five composite measures encompassing functional engagement were not associated with cognitive change.

**Conclusion:**

These findings underscore that increasing structural engagement alone is insufficient for protecting cognitive health, emphasizing the critical importance of relationship quality. Negative relationships may place greater cognitive demands than positive ones, fostering increased cognitive reserve and supporting the cognitive stimulation hypothesis. In contrast, positive relationships showed limited impact, aligning with the lack of support for the social support hypothesis. However, the detrimental effects of negative family relationships warrant further investigation, as they may contribute to cognitive decline through mechanisms such as chronic stress or emotional overload.

